# Supplementary material for: Approaching coupled cluster accuracy with a general-purpose neural network potential through transfer learning
Source: Nat Commun. 2019 Jul 1;10:2903. doi: 10.1038/s41467-019-10827-4 (PMC6602931; doi:10.1038/s41467-019-10827-4)
Supplement: Supplementary file 1 — Supplementary information [file 41467_2019_10827_MOESM1_ESM.pdf]

# Supplementary information

# Approaching coupled cluster accuracy with a general-purpose neural network potential through transfer learning

**Smith et al.**

## Supplementary Methods

### S1.1 Detailed description of the CCSD(T)*/CBS scheme

The linear-scaling domain-localized DPLNO approximation, and its accuracy, are primarily controlled by an electron pair cutoff threshold. There are three pre-set thresholds implemented in ORCA: LoosePNO, NormalPNO, and TightPNO, which are recommended for rapid estimation, general thermochemistry and kinetics, and non-covalent interactions and molecular conformations, respectively.[1] They were benchmarked against two high-quality and distinct datasets: S66[2] for intermolecular interaction energies relevant to biomolecules and FH51[3] for reaction energies of medium-sized organic molecules. The effect of tightening the DPLNO threshold is very significant for these datasets: TightPNO setting reduces energy RMSD from 0.36 to 0.06 kcal*mol^-1 for S66 and from 0.38 to 0.16 kcal*mol^-1 for HF51, compared to NormalPNO.[1] Therefore, in order to obtain a high-quality approximation to CCSD(T) energies TightPNO thresholds must be properly chosen.

Another key component of our efficient computational method is the complete basis set (CBS) extrapolation. We have applied the widely used two-point "EP1"[4] extrapolation scheme originally proposed by Hobza and coworkers[5]. The total energy is computed as the sum of the MP2/CBS extrapolated energy and the difference between CCSD(T) and MP2 energies calculated with a smaller basis set. For the sake of computational efficiency we have used cc-pVTZ and cc-pVQZ basis sets for extrapolation of HF and MP2 energies using the formulas of Halkier[6] and Helgaker[7] ($\alpha_{34}$ = 5.46, $\beta_{34}$ = 3.05 [8]). The difference between CCSD(T) and MP2 energies was estimated with the cc-pVTZ basis set as shown in Supplementary Equation 1.

$$E_{total}^{CBS} \approx E_{HF}^{CBS} + E_{MP2}^{CBS} + \left(E_{CCSD(T)}^{cc-pVTZ} - E_{MP2}^{cc-pVTZ}\right) \quad 1$$

The most computationally demanding term in the Equation 1 is $E_{CCSD(T)}^{cc-pVTZ}$, which we need to calculate with at least TightPNO level of accuracy. To reduce computational cost further, we approximated this term using an idea similar to the CBS extrapolation approach above: TightPNO and NormalPNO-CCSD(T) energies were calculated with a smaller basis set and the difference was added to the NormalPNO-CCSD(T)/cc-pVTZ energy. Summarizing, the CCSD(T)/cc-pVTZ part in Supplementary Eq. 1 was estimated with Supplementary Equation 2.

$$E_{CCSD(T)}^{cc-pVTZ} \approx E_{Normal-DPLNO-CCSD(T)}^{cc-pVTZ} + (E_{Tight-DPLNO-CCSD(T)}^{cc-pVDZ} - E_{Normal-DPLNO-CCSD(T)}^{cc-pVDZ}) \quad 2$$

Such a composite scheme for approximating CCSD(T)/CBS energies is computationally efficient, as it requires computation of Tight-DPLNO-CCSD(T) with at most a double-zeta basis set. The robustness of this suggested CCSD(T)*/CBS scheme is evaluated against two distinct datasets with high-quality CCSD(T)-F12 data available: S66 benchmark[9] for weak intermolecular interactions and the extensive W4-11 benchmark[10] for thermochemistry. CCSD(T)*/CBS performs excellent on both benchmarks (See Supplementary Table 1).

In Supplementary Table 1, the extrapolation schemes are noted with the “small” basis set, which is used to calculate ΔCCSD(T) with respect to MP2, the MP2/CBS energy obtained using extrapolation with basis set with higher cardinal number. CCSD(T)*/CBS is equal to the TightPNO-CCSD(T)*/CBS(TZ) scheme, which was applied for obtaining our reference training dataset. TightPNO-CCSD(T)* denotes that the ΔTightPNO term is the difference between TightPNO and NormalPNO energy using a basis set with lower cardinal number. The data in Supplementary Table 1 shows that CCSD(T)*/CBS provides excellent balance between cost and performance with errors no worse than the canonical CCSD(T)/CBS(aDZ) method. It also should be noted that incorporating the ΔTightPNO approach helps to achieve much lower error, compared to NormalPNO, without sacrificing much computational efficiency.

### S1.2 Neural network model details

#### S1.2.1 Neural network architecture

The models trained in this work all utilize variable size networks for different atomic species. This is done since the molecules in the ANI-1x datasets are proportioned 48% H, 30% C, 13% N, and 9% O. Supplementary Table 2 provides details of each model architecture used in this work.

#### S1.2.2 Atomic environment vector parameters

The ANI model atomic environment vector (AEV) is computed using the in-house NeuroChem software suite. These AEVs are computed identically to those published in the ANI-1 work.[11] In this work the atomic elements C, H, N, and O are described by the AEVs (using the parameters below) yielding a total of 384 AEV elements per atom. The AEV parameters used to train each model are supplied below.

Radial Parameters:

- Radial cutoff = 5.2 Å

- $\eta^{Radial}$ = [16]
- $R_s^{Radial} = [0.900000, 1.168750, 1.437500, 1.706250, 1.975000, 2.243750, 2.51250, 2.781250, 3.050000, 3.318750, 3.587500, 3.856250, 4.125000, 4.39375, 4.662500, 4.931250]$ Å

Angular Parameters:

- Angular cutoff = 3.5 Å
- $\eta^{Angular}$ = [8]
- $R_s^{Radial} =$ [0.900000, 1.550000, 2.200000, 2.850000] Å
- $\zeta =$ [32.00000]
- $\theta_s = [0.19634954, 0.58904862, 0.9817477, 1.3744468, 1.7671459, 2.1598449, 2.552544, 2.945243]$

### S1.2.3 Training details

Prior to training the ANI models, a linear fitting to the energy per atomic species is performed—essentially, an empirical self-energy term for each element. This linear fitting is over the entire dataset and the fit is performed with respect to the number of each atomic element in a given molecule as the input. The ANI models are then trained to the QM calculated energy minus the linear fitted prediction. The energy obtained from this process is roughly analogous to the process of computing an atomization energy, but without any per-atom bias. The linear fitting parameters (in Hartrees) used in this work are provided below.

ANI-1x DFT Linear fitting parameters:

- H = -0.600952980000
- C = -38.08316124000
- N = -54.70775770000
- O = -75.19446356000

ANI-1x CCSD(T)*/CBS Linear fitting parameters:

- H = -0.5991501324919538
- C = -38.03750806057356
- N = -54.67448347695333
- O = -75.16043537275567

ANI-1x DFT to CCSD(T)*/CBS Δ Linear fitting parameters:

- H = -0.003172990955487249
- C = 0.04396089482092749
- N = 0.03789128635905942
- O = 0.029194038876402876

The following hyperparameters are used during training for all ANI models. These parameters have been determined through rigorous hyperparameter searches in prior ANI potential[11,12] development.

- Mini-batch size: 2560 molecules
- Initial learning rate: 1.0E-3
- Patience for annealing learning rate: 100
- Multiplier for annealing learning rate: 0.5
- Learning rate for training termination: 1.0E-5
- ADAM[13] stochastic optimization is used with default parameters

A single epoch of training the ANI-1x DFT models takes 12.5s while a single epoch for training the ANI-1x CCSD(T)*/CBS model takes 1.25s on a single Titan V. Approximately 1450 epochs of training are carried out on all models before convergence. This leads to a total training time of 5 hours for the DFT models, 5.5 hours for training the DFT plus transfer and Δ-learning models, and 0.5 hours for training the model which was only trained to the CCSD(T)*/CBS data.

### S1.2.4 Ensemble held out test set results

Supplementary Table 3 provides the 1/8th held out test set mean absolute error (MAE) and root mean squared error (RMSE) for the DFT trained ANI-1x and CCSD(T)*/CBS trained ANI-1ccx and ANI-1ccx-R single models from the ensemble. The ANI-1ccx models were trained to a small dataset (500k) still fits to its reference as well as the ANI-1x models trained to a larger dataset (5M). However, the ANI-1ccx-R models, which were only trained to the coupled cluster data with no transfer learning, performs significantly worse on the held-out test set. From this we conclude that transfer learning is performing as designed by providing the performance of a model trained to 5M datapoints with only 500k datapoints.

## S1.3 Active learning molecular torsions

Since molecular dynamics simulations and normal mode perturbation is used for sampling the ANI-1x dataset, molecular torsion barriers can be poorly described by the ANI-1x potential. In other words, ANI torsion barriers tend to have higher error than near equilibrium conformations due to the sampling methods used to generate training data. To reduce this error and improve barrier sampling, we develop an iterative and entirely ML driven active learning technique for automatically sampling molecular torsions. We begin with a dataset of SMILES [opensmiles.org] strings; in this work we start with a portion of the ChEMBL[14–16] database containing less than 20 total atoms. We also include the SMILES strings for molecules in the Genentech torsion benchmark[17] since these molecules represent a diverse set of dihedrals in the simplest chemical environment that they can be found. From the resulting set of SMILES strings we carry out active learning iterations as follows:

1. Randomly select N smiles from the database
2. Embed the N molecules in 3D space

3. Randomly select a rotatable bond, and direction of rotation
4. Conduct relaxed scan every 10 degrees (36 points) of each selected torsion using the current ANI model
5. Carry out an ensemble disagreement test (see our work on active learning for details; a selection criterion of $\hat{\rho} = 0.2$ kcal*mol^-1 is used in this work)[12] on the 36 * N generated molecular conformations
6. For all M conformations that fail the ensemble disagreement test, compute ANI normal modes
7. Randomly perturb the M conformations along the normal modes to a maximum distance of 0.2Å along each mode. Generate 4 normal mode sampled (NMS) points for each M
8. Generate DFT energies and forces for all NMS points
9. Add resulting data to the training dataset and retrain ANI model
10. Go back to 1 and iterate

We complete 20 iterations of the above scheme resulting in the generation of 202k extra DFT datapoints. We subsample 19k points from this dataset generation using our CCSD(T)/CBS extrapolation scheme. Supplementary Figure 1 compares ANI-1ccx and ANI-1x with and without the active learning generated dihedral corrections.

## Supplementary Tables

**Supplementary Table 1**: Computational efficiency for example small molecules and performance of the CCSD(T)/CBS approximation (CCSD(T)*/CBS) evaluated against CCSD(T)-F12 reference data and compared to other composite schemes.

| **Method** | **CPU-core hours[a]** | | **MAE / RMSD, kcal*mol^-1** | |
|---|---|---|---|---|
| | Alanine | Aspirin | S66[b] | W4-11[c] |
| CCSD(T)/CBS(aDZ) | 1.53 | 42.79 | 0.08 / 0.10 | 1.58 / 1.85 |
| CCSD(T)/CBS(haTZ) | 9.13 | 427.00 | 0.03 / 0.04 | 1.31 / 1.53 |
| NormalPNO-CCSD(T)/CBS(aDZ) | 0.78 | 4.63 | 0.31 / 0.39 | 2.35 / 2.59 |
| NormalPNO-CCSD(T)/CBS(haTZ) | 1.85 | 16.83 | 0.27 / 0.36 | 1.91 / 1.66 |
| TightPNO-CCSD(T)/CBS(TZ) | 1.56 | 16.70 | 0.16 / 0.10 | 1.40 / 1.50 |
| CCSD(T)*/CBS (our reference) | 1.44 | 7.44 | 0.09 / 0.10 | 1.46 / 1.55 |

[a] Calculations performed on an Intel(R) Xeon(R) E5-2630 v3 @ 2.40GHz CPU
[b] Reference data from [9]
[c] Reference data from [10], averaged for all subsets of W4-11.
[d] Original reference data from [2]
[e] Revised S66 data from [18]

**Supplementary Table 2**: Fully connected neural network architectures for each atom type.

| Hydrogen Network Architecture | | | | |
|---|---|---|---|---|
| | **Layer1** | **Layer2** | **Layer3** | **Layer4** |
| **Nodes** | 160 | 128 | 96 | 1 |
| **Activation** | CELU[19] | CELU | CELU | Linear |
| **Regularization** | MAX NORM (1.0E-4) | MAX NORM (1.0E-5) | MAX NORM (1.0E-6) | None |

| Carbon Network Architecture | | | | |
|---|---|---|---|---|
| | **Layer1** | **Layer2** | **Layer3** | **Layer4** |
| **Nodes** | 144 | 112 | 96 | 1 |
| **Activation** | CELU | CELU | CELU | Linear |
| **Regularization** | MAX NORM (1.0E-4) | MAX NORM (1.0E-5) | MAX NORM (1.0E-6) | None |

| Oxygen and Nitrogen Network Architecture | | | | |
|---|---|---|---|---|
| | **Layer1** | **Layer2** | **Layer3** | **Layer4** |
| **Nodes** | 128 | 112 | 96 | 1 |
| **Activation** | CELU | CELU | CELU | Linear |
| **Regularization** | MAX NORM (1.0E-4) | MAX NORM (1.0E-5) | MAX NORM (1.0E-6) | None |

**Supplementary Table 3**: ANI held out test set performance. In the case of ANI-1x the errors are with respect to the reference DFT, while for ANI-1ccx and ANI-1ccx-R the errors are with respect to the CCSD(T)*/CBS reference.

| **Model ID** (from 8x ensemble) | **ANI-1x test set performance** | | **ANI-1ccx test set performance** | | **ANI-1ccx-R test set performance** | |
|---|---|---|---|---|---|---|
| | **MAE** | **RMSE** | **MAE** | **RMSE** | **MAE** | **RMSE** |
| **1** | 1.75 | 2.55 | 1.78 | 2.54 | 2.30 | 3.35 |
| **2** | 1.78 | 2.60 | 1.82 | 2.80 | 2.26 | 3.30 |
| **3** | 1.77 | 2.58 | 1.78 | 2.63 | 2.26 | 3.26 |
| **4** | 1.73 | 2.53 | 1.77 | 2.55 | 2.24 | 3.23 |
| **5** | 1.76 | 2.66 | 1.76 | 2.51 | 2.24 | 3.25 |
| **6** | 1.73 | 2.54 | 1.75 | 2.50 | 2.26 | 3.26 |
| **7** | 1.75 | 2.62 | 1.76 | 2.47 | 2.24 | 3.34 |
| **8** | 1.76 | 2.61 | 1.79 | 2.59 | 2.21 | 3.20 |
| **Mean** | **1.75** | **2.59** | **1.78** | **2.57** | **2.25** | **3.27** |

**Supplementary Table 4.** Computed conformer ΔE on the GDB-10to13 benchmark (all conformers within 100 kcal*mol^-1 of the global minimum) for the transfer learning-based ANI-1ccx and the Δ-learning based ANI-1ccx-Δ potential. Uncertainties are the standard deviation of each ANI model's error from the ANI ensemble used for the mean prediction. Energy units are kcal*mol^-1.

| | ANI-1ccx | ANI-1ccx-Δ |
|---|---|---|

| | | |
|---|---|---|
| **MAD** | 1.46±0.02 | 1.44±0.04 |
| **RMSD** | 2.07±0.04 | 2.04±0.15 |

**Supplementary Table 5**. GDB-10to13 benchmark results comparing various ANI potentials and DFT. Errors for conformer energy differences ($\Delta\mathbf{E}$) and potential energies ($\mathbf{E}$) for all ANI potentials. ωB97X/6-31G* errors are also provided. The blank cells are for values which cannot be compared because absolute energy differences between DFT and CCSD(T)*/CBS are arbitrary. μ and σ are the arithmetic mean and standard deviation, respectively. M and R are the MAE and RMSE, respectively. Units of energy are kcal*mol^-1.

| **Model** | $\Delta\mathbf{E}_\mathbf{M}^\mu$ | $\Delta\mathbf{E}_\mathbf{M}^\sigma$ | $\Delta\mathbf{E}_\mathbf{R}^\mu$ | $\Delta\mathbf{E}_\mathbf{R}^\sigma$ | $\mathbf{E}_\mathbf{M}^\mu$ | $\mathbf{E}_\mathbf{M}^\sigma$ | $\mathbf{E}_\mathbf{R}^\mu$ | $\mathbf{E}_\mathbf{R}^\sigma$ |
|---|---|---|---|---|---|---|---|---|
| ANI-1x | 3.84 | 0.07 | 5.82 | 0.12 | -- | -- | -- | -- |
| ANI-1ccx | 2.24 | 0.02 | 3.24 | 0.04 | 2.22 | 0.05 | 3.02 | 0.04 |
| ANI-1ccx-Δ | 2.21 | 0.07 | 3.18 | 0.10 | -- | -- | -- | -- |
| ANI-1ccx-R | 2.77 | 0.05 | 3.97 | 0.08 | 2.72 | 0.08 | 3.65 | 0.08 |
| ωB97X/6-31G* | 3.37 | -- | 4.99 | -- | -- | -- | -- | -- |

**Supplementary Table 6:** Performance of the two target methods and two ANI potentials used in this work. The performance comparison is on the HC7 hydrocarbon reaction energy benchmark and the ISOL6 organic molecule isomerization energy benchmark.

| | ***CCSD(T)*/CBS*** | ***ANI-1ccx*** | ***ωB97X/6-31g(d)*** | ***ANI-1x*** |
|---|---|---|---|---|
| ***HC7*** | 1.6/1.8 | 2.5/2.9 | 16.4/22.2 | 19.1/24.6 |
| ***ISOL6*** | 0.5/0.5 | 1.5/1.8 | 3.8/4.7 | 4.6/5.3 |

**Supplementary Table 7:** Reference and calculated energies (kcal*mol^-1) for the HC7/11 benchmark. Reference calculations are obtained from Peverati, Zhao, and Truhlar [a]. The rows of the table correspond to the index given in the HC7/11 portion of Figure 3 in the main article.

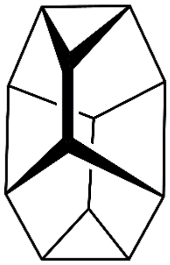

E1 (1)

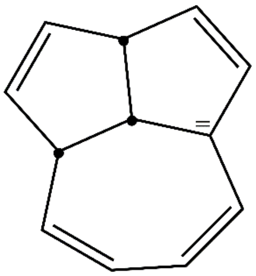

E2 (22)

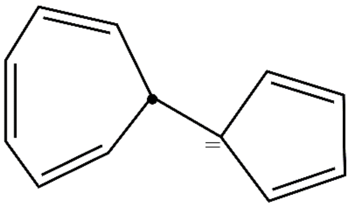

E3 (31)

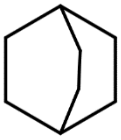

E4
**(Bicyclo[2.2.2]octane)**

| **Reaction details** | **Reference[a]** | **ANI-1ccx** | **CCSD(T)*/CBS** | **ANI-1x** | ***ω*B97X/6-31g(d)** |
|---|---|---|---|---|---|
| E1 → E2 | 14.34 | 15.84 | 13.72 | 36.31 | 28.77 |
| E1 → E3 | 25.02 | 30.71 | 23.23 | 54.43 | 41.09 |
| Octane-a → Octane-b | 1.90 | 0.22 | -1.36 | 2.79 | 1.75 |
| $4CH_4 + C_6H_{14} \rightarrow 5C_2H_6$ | 9.81 | 7.72 | 8.74 | 7.00 | 6.26 |
| $6CH_4 + C_8H_{18} \rightarrow 7C_2H_6$ | 14.84 | 11.67 | 13.09 | 10.49 | 9.30 |
| Adamantane → $3CH_4 + 2C_2H_2$ | 193.99 | 196.48 | 196.27 | 237.51 | 238.83 |
| E4 → $3CH_4 + 2C_2H_2$ | 127.22 | 127.86 | 127.43 | 158.05 | 157.65 |

[a] Reference data from[20]

**Supplementary Table 8:** Reference and calculated energies (kcal*mol^-1) for the ISOL6[a] benchmark. Reference calculations are obtained from Peverati, Zhao, and Truhlar [b]. The rows of the table correspond to the index give in the ISOL6 portion of Figure 3 in the main article. One reaction involving the atomic element fluorine (F) was left out since the ANI potential in this work is only fit to CHNO.

**1** (3)

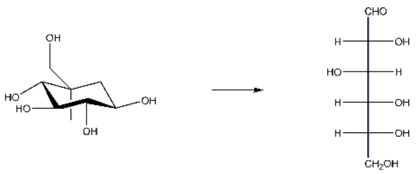

**2** (9)

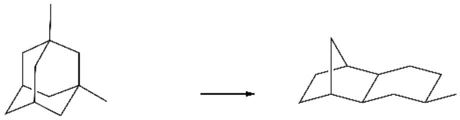

**3** (10)

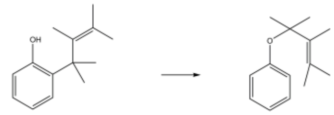

**4** (13)

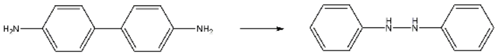

**5** (14)

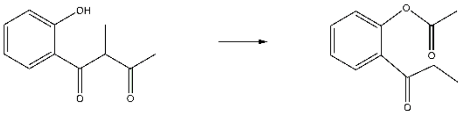

| Reaction | Reference[a] | ANI-1ccx | CCSD(T)*/CBS | ANI-1x | *ω*B97X/6-31g(d) |
|---|---|---|---|---|---|
| **1** | 9.77 | 9.16 | 10.45 | 6.45 | 9.32 |
| **2** | 21.76 | 21.48 | 21.07 | 20.85 | 20.80 |
| **3** | 6.82 | 3.82 | 6.39 | -2.18 | 1.03 |
| **4** | 33.52 | 35.36 | 33.30 | 27.84 | 26.43 |
| **5** | 5.30 | 6.87 | 5.62 | 1.06 | 0.40 |

[a] Reference data from[21]
[b] Reference data from[20]

**Supplementary Figures**

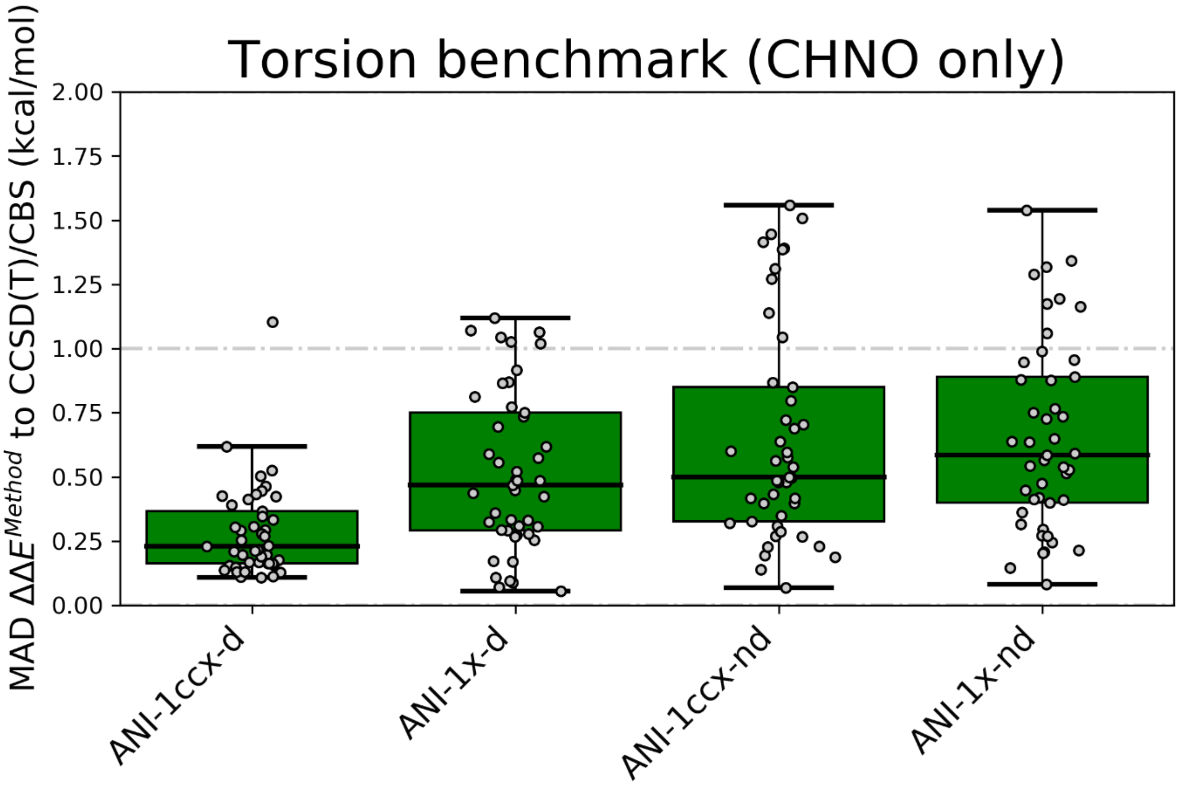


**Supplementary Figure 1**: Comparison of ANI model performance with and without transfer learning and dihedral active learning reparameterization on 45 torsion profiles containing the atomic elements C, H, N, and O. The ANI potentials with '-d' appended are reparametrized for better torsions and with '-nd' represent no reparameterization data was used during training. The grey dots represent the MAD of a given torsion scan vs. gold standard CCSD(T)/CBS. The box extends from the upper to lower quartile of the MAD distribution, the black horizontal line in the box is the median MAD. All ANI methods carried out using restrained optimization with ANI forces.

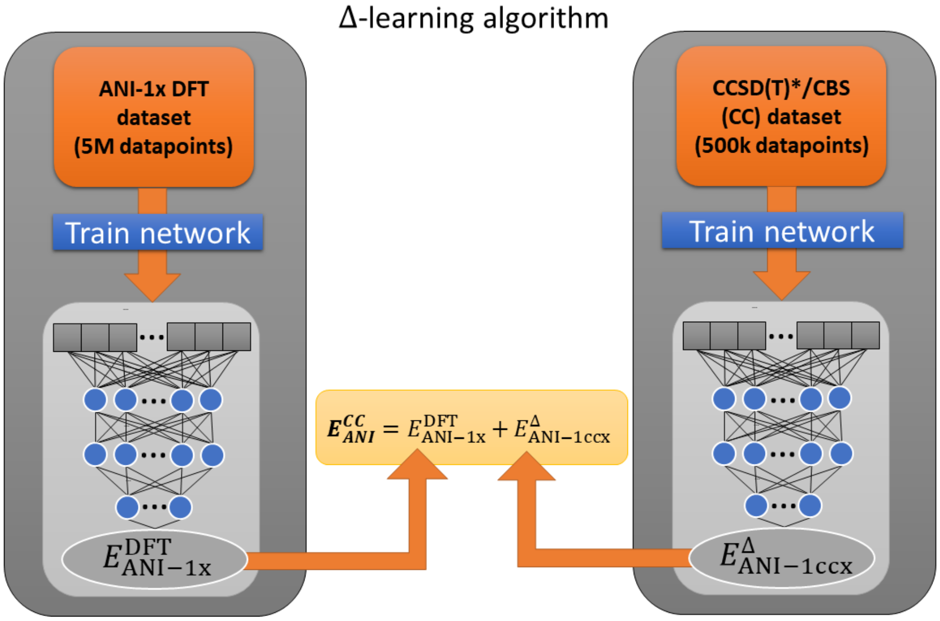


**Supplementary Figure 2**: Diagram of the delta learning techniques evaluated in this work. Δ-learning uses the pretrained ANI DFT model to predict an energy, then a second network is trained to correct the DFT prediction to better predict the CCSD(T)/CBS-extrapolated data. The resulting scheme requires two networks for prediction and is thus more expensive than the transfer learning.

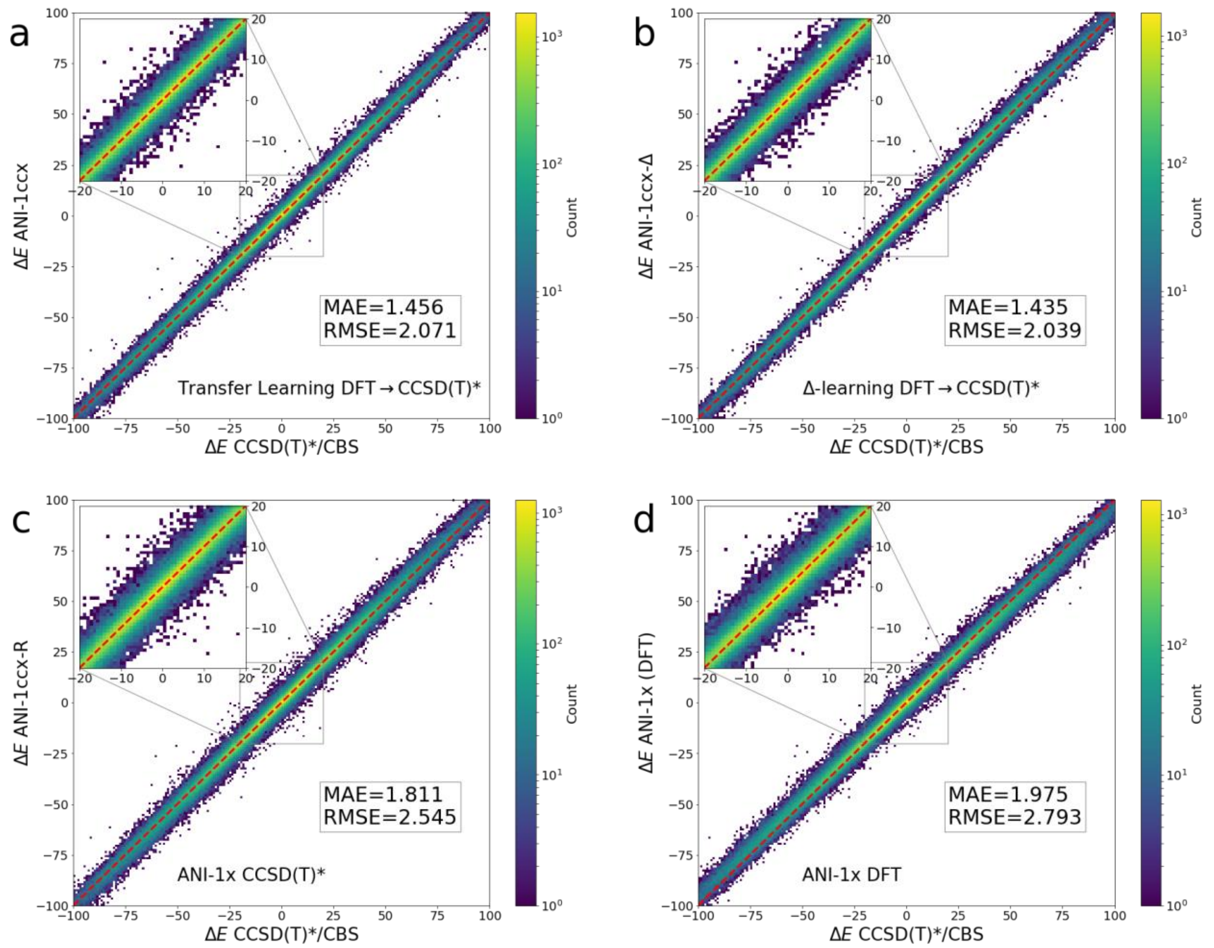


**Supplementary Figure 3**: Comparison of four different ANI potentials at predicting relative energies (ΔE) between all conformers for each molecule in the GDB-10to13 (CCSD(T)/CBS* computed) benchmark. These log-scale density correlation plots show a) ANI-1ccx (transfer learning-based CCSD(T)*/CBS trained model), b) ANI-1ccx-Δ (Δ-learning based CCSD(T)*/CBS trained model), c) ANI-1ccx-R (model trained only to the CCSD(T)*/CBS dataset), and d) ANI-1x (DFT trained model).

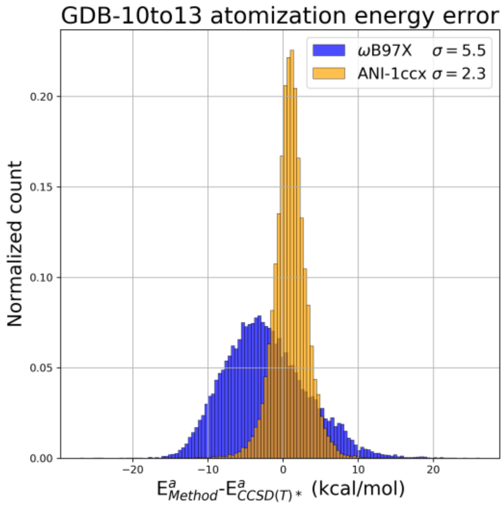


**Supplementary Figure 4:** Per atom corrected atomization energy difference ($\Delta E^a$) distribution for ωB97X/6-31G* and ANI-1ccx vs. CCSD(T)*/CBS reference data. An average correction of 18.5 kcal*mol^-1 was applied to the DFT data so it better fits the CCSD(T)*/CBS atomization energies. This correction came from a non-trivial linear fitting to the atomization energy difference between DFT and CCSD(T)*/CBS, based on the number of each atomic element. No such correction was applied to the ANI-1ccx data.

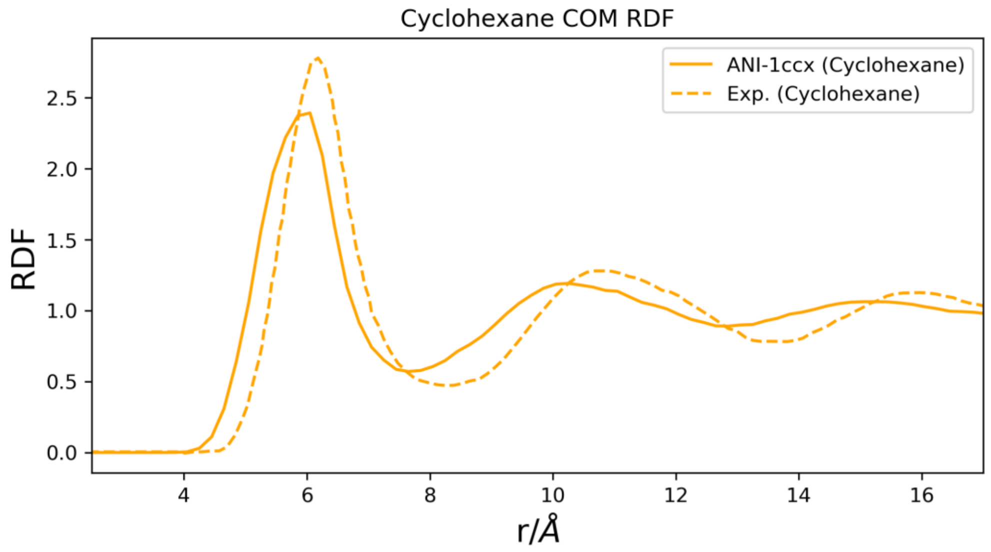


**Supplementary Figure 5:** Comparison of C-C center of mass radial distribution functions (RDF) from experiment[22] and ANI-1ccx. Simulations was carried out with the NVT ensemble using the Langevin thermostat set to 300K.

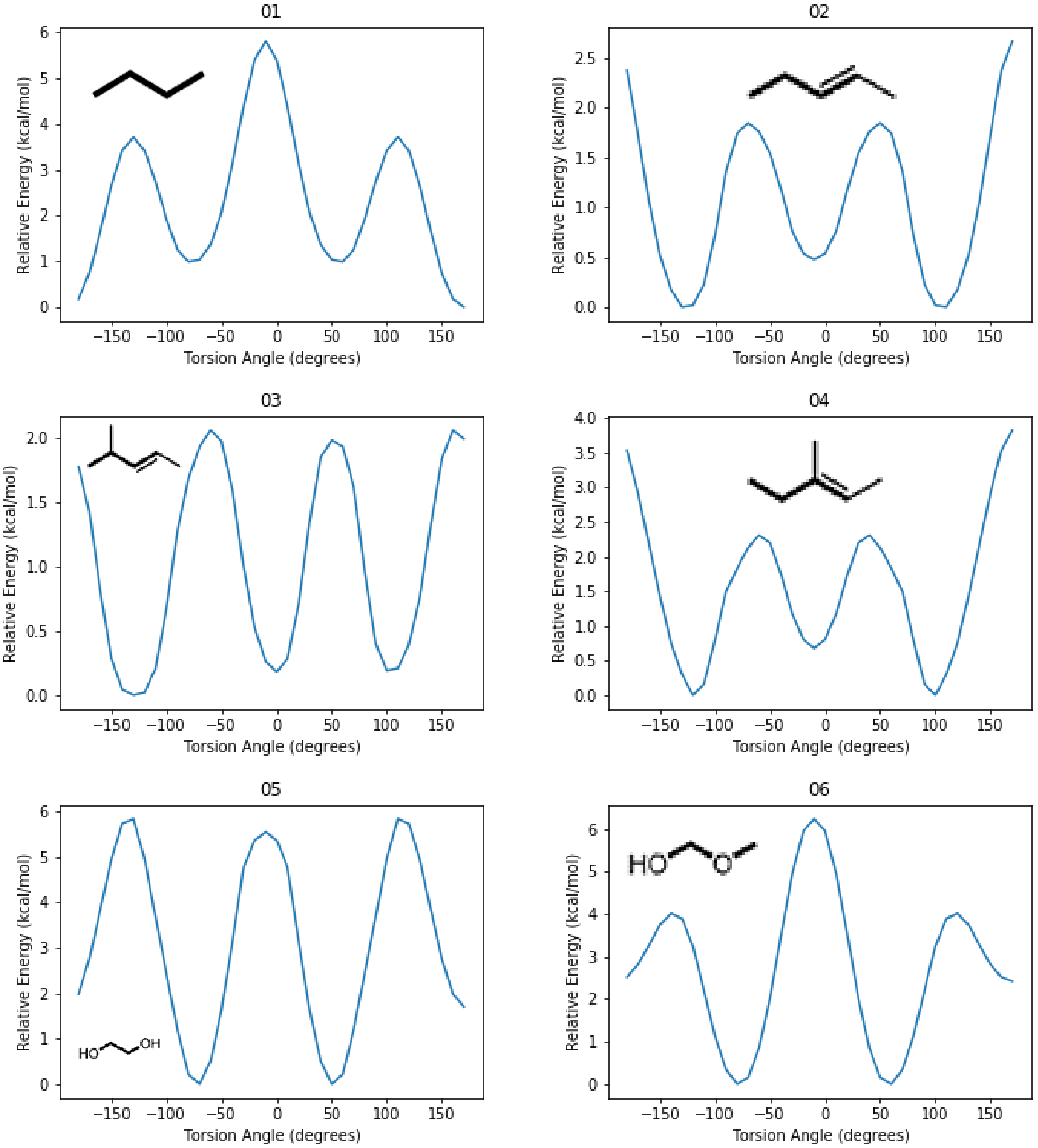
01
02
03
04
05
06
Relative Energy (kcal/mol)
Torsion Angle (degrees)
HO
OH
HO
O

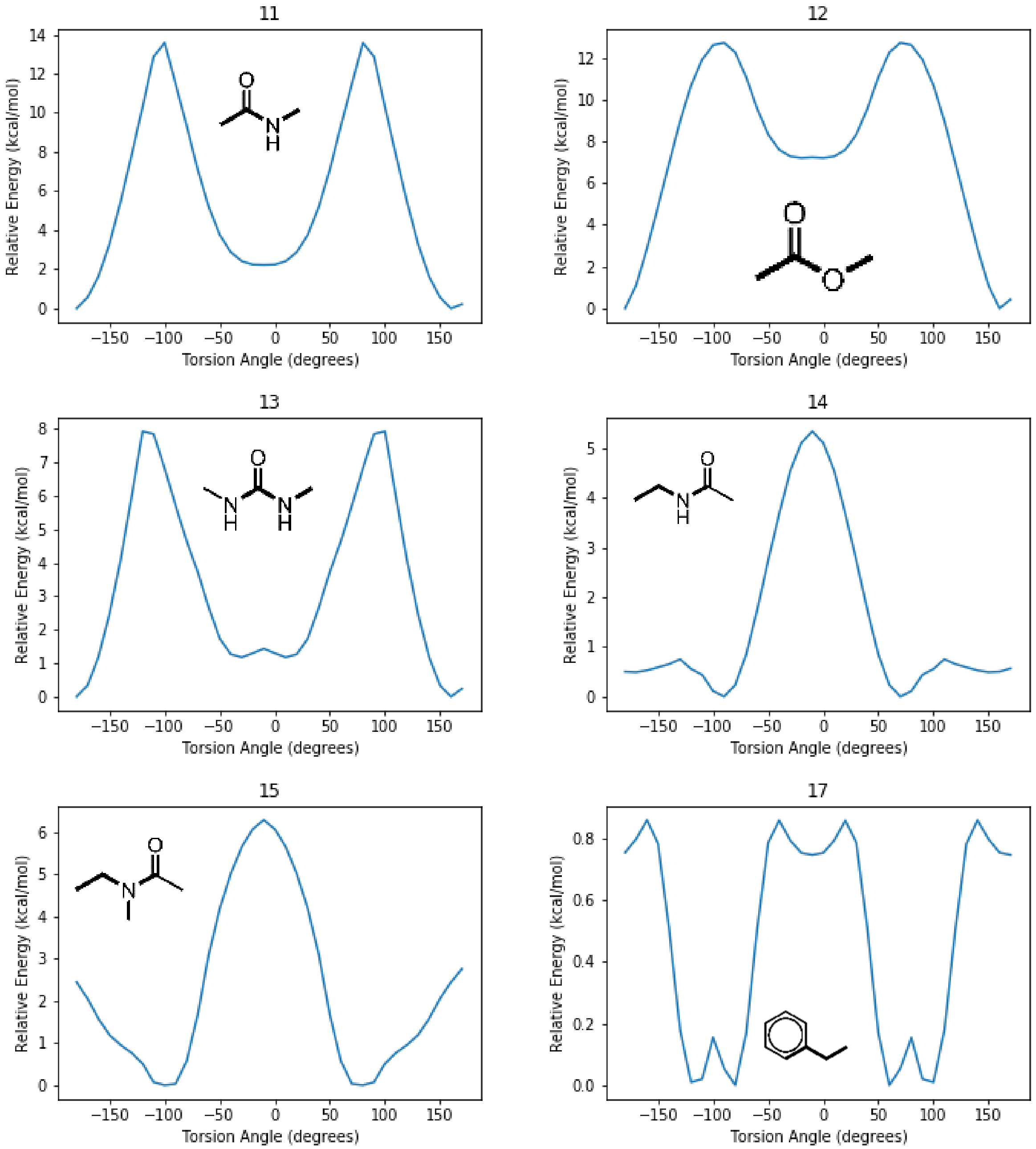
11
12
13
14
15
17
Relative Energy (kcal/mol)
Torsion Angle (degrees)

18

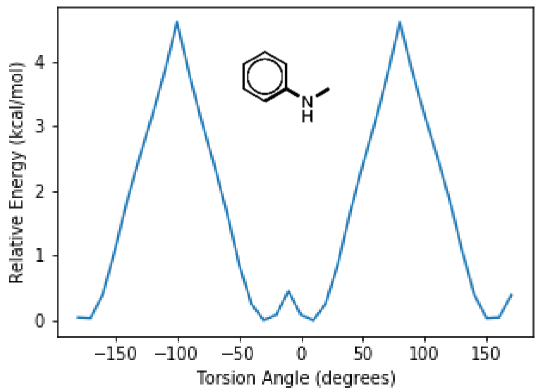


19

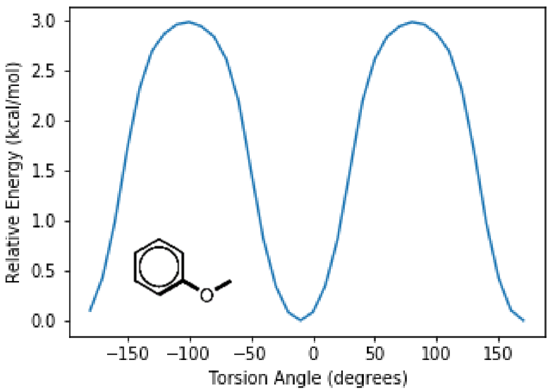


20

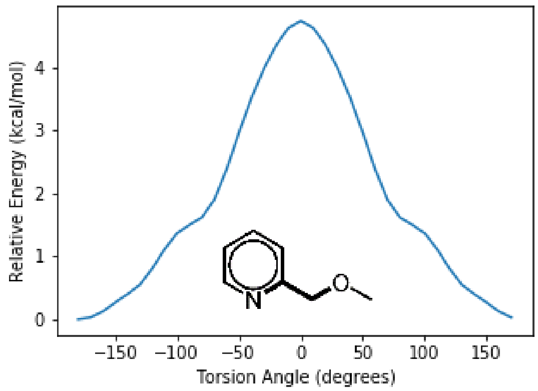


21

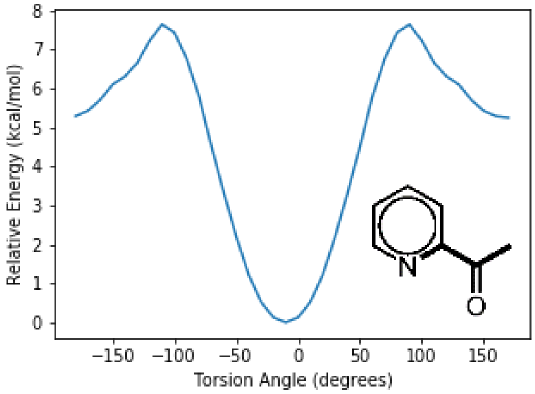


22

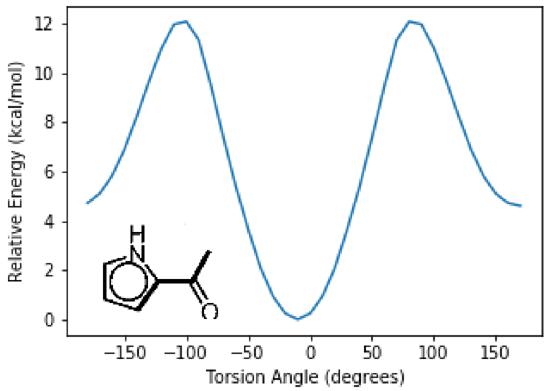


23

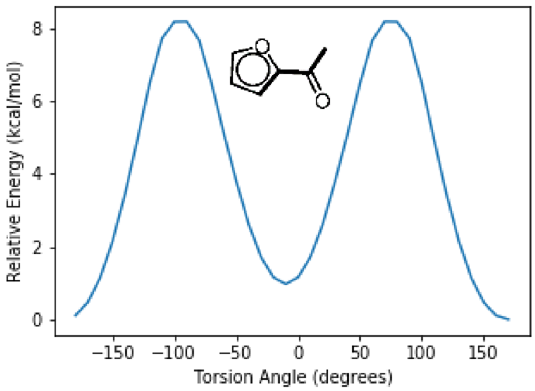

25

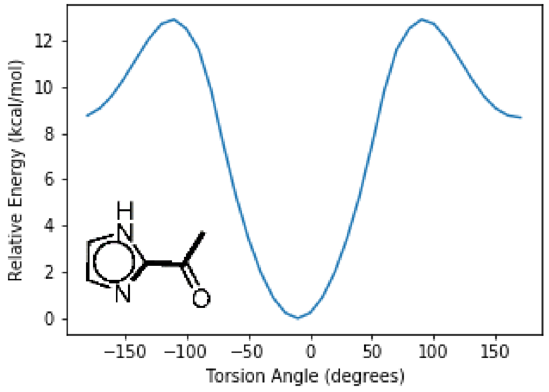


26

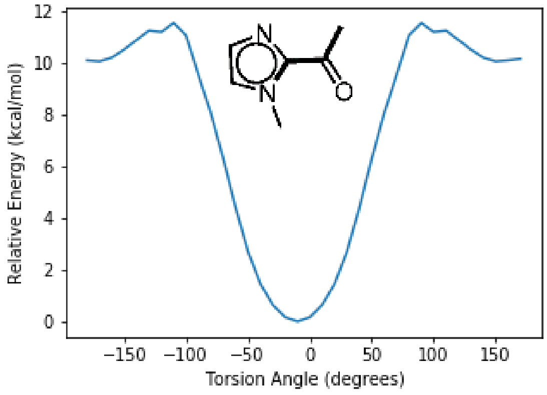


27

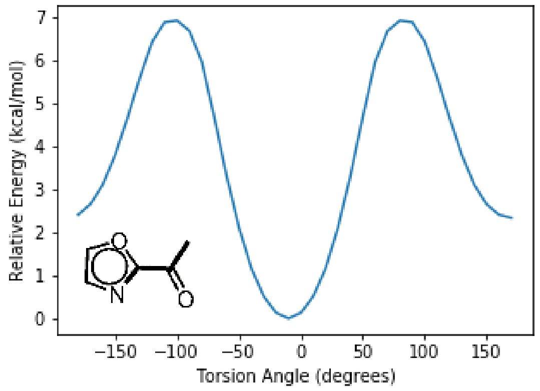


31

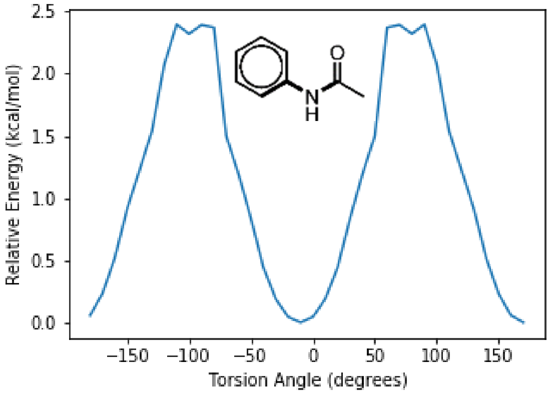


32

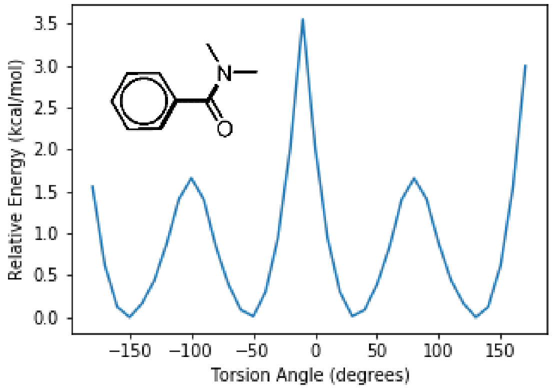


33

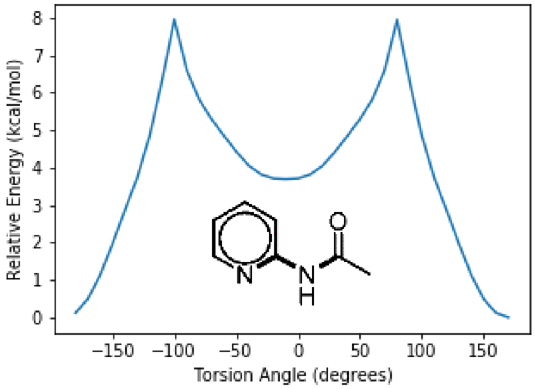

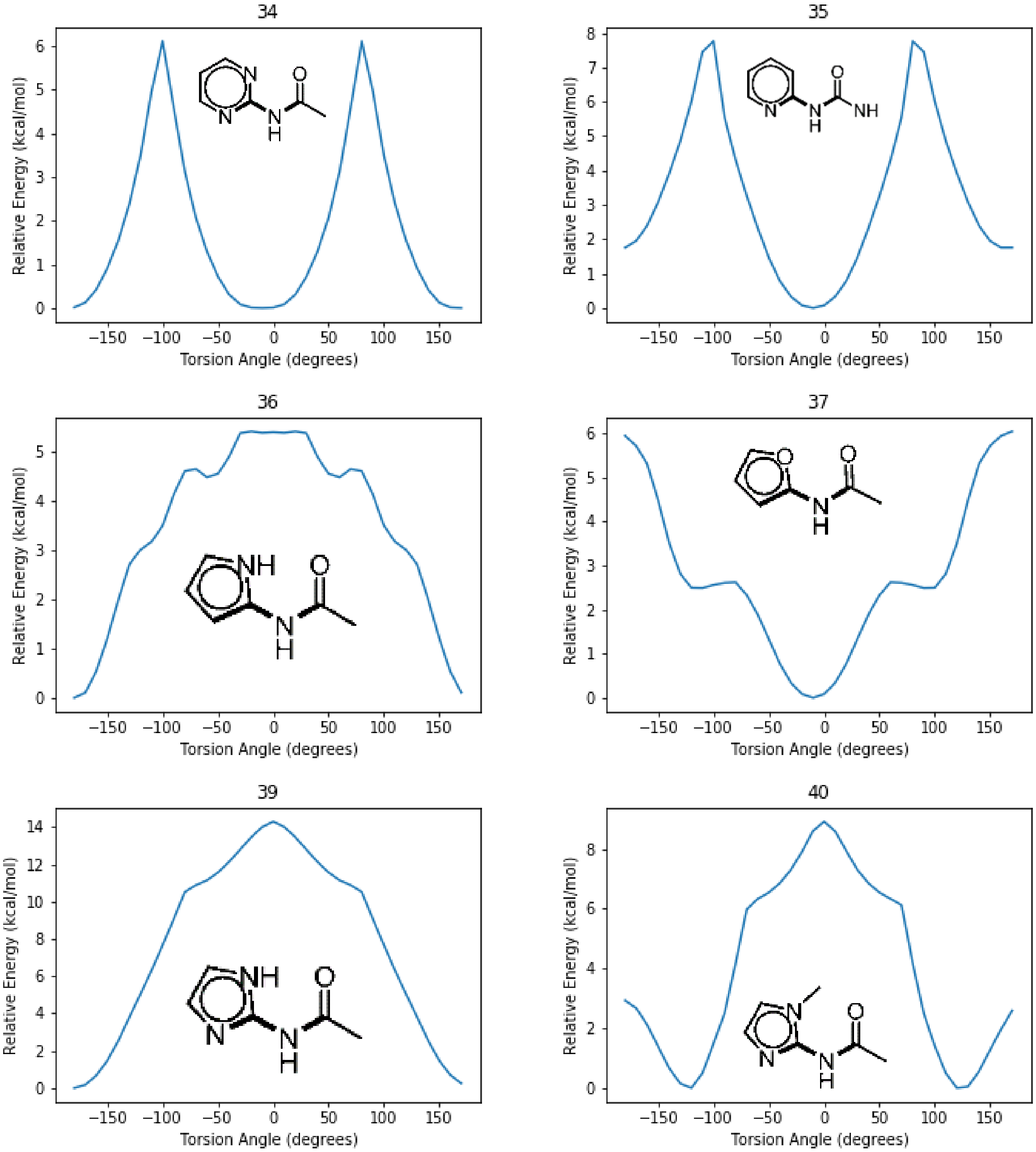
34
35
36
37
39
40
Relative Energy (kcal/mol)
Torsion Angle (degrees)
−150
−100
−50
0
50
100
150
N
O
NH
H

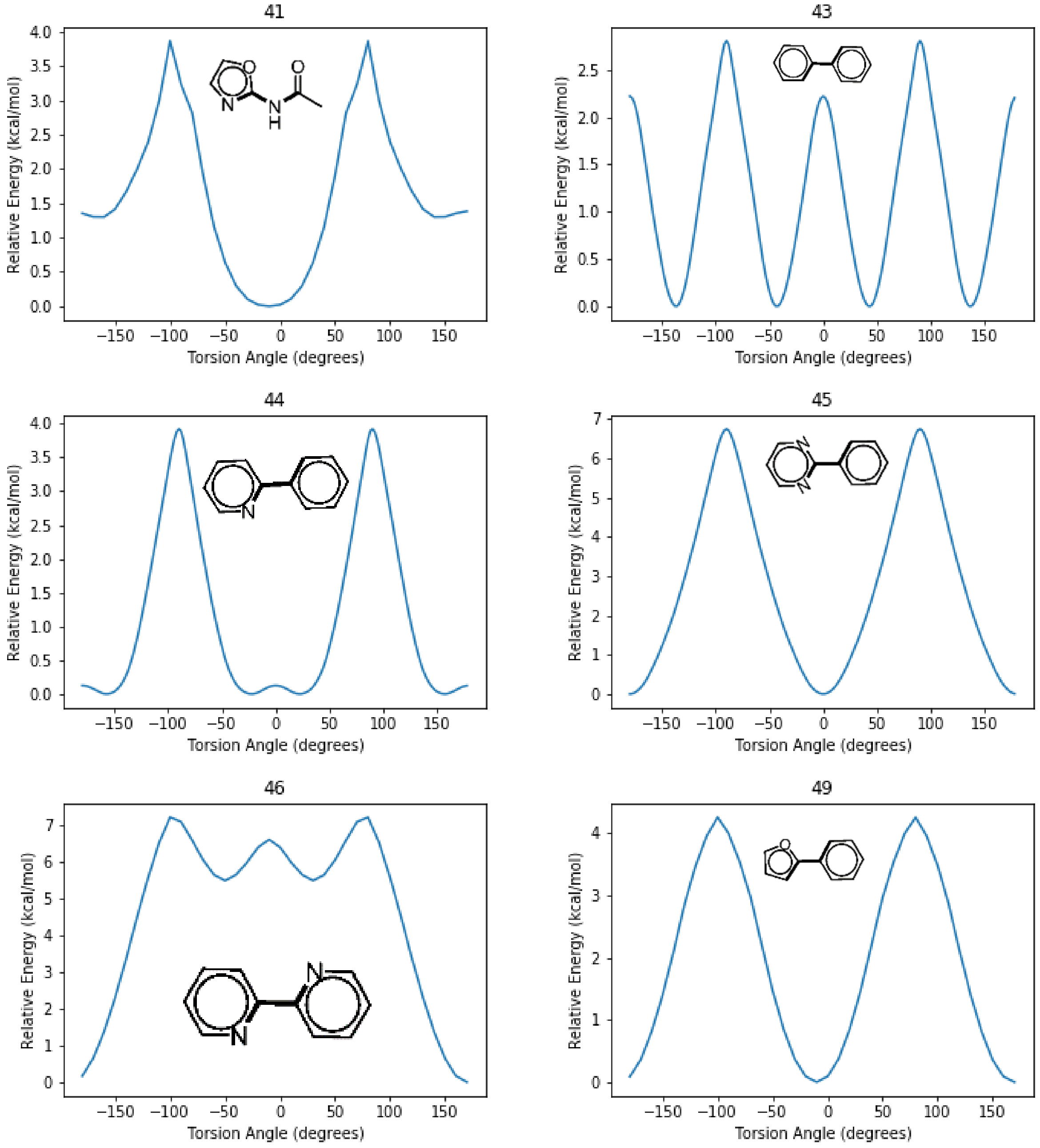
41
43
44
45
46
49
Relative Energy (kcal/mol)
Torsion Angle (degrees)
O
N
N
H
N
N
N
N
N

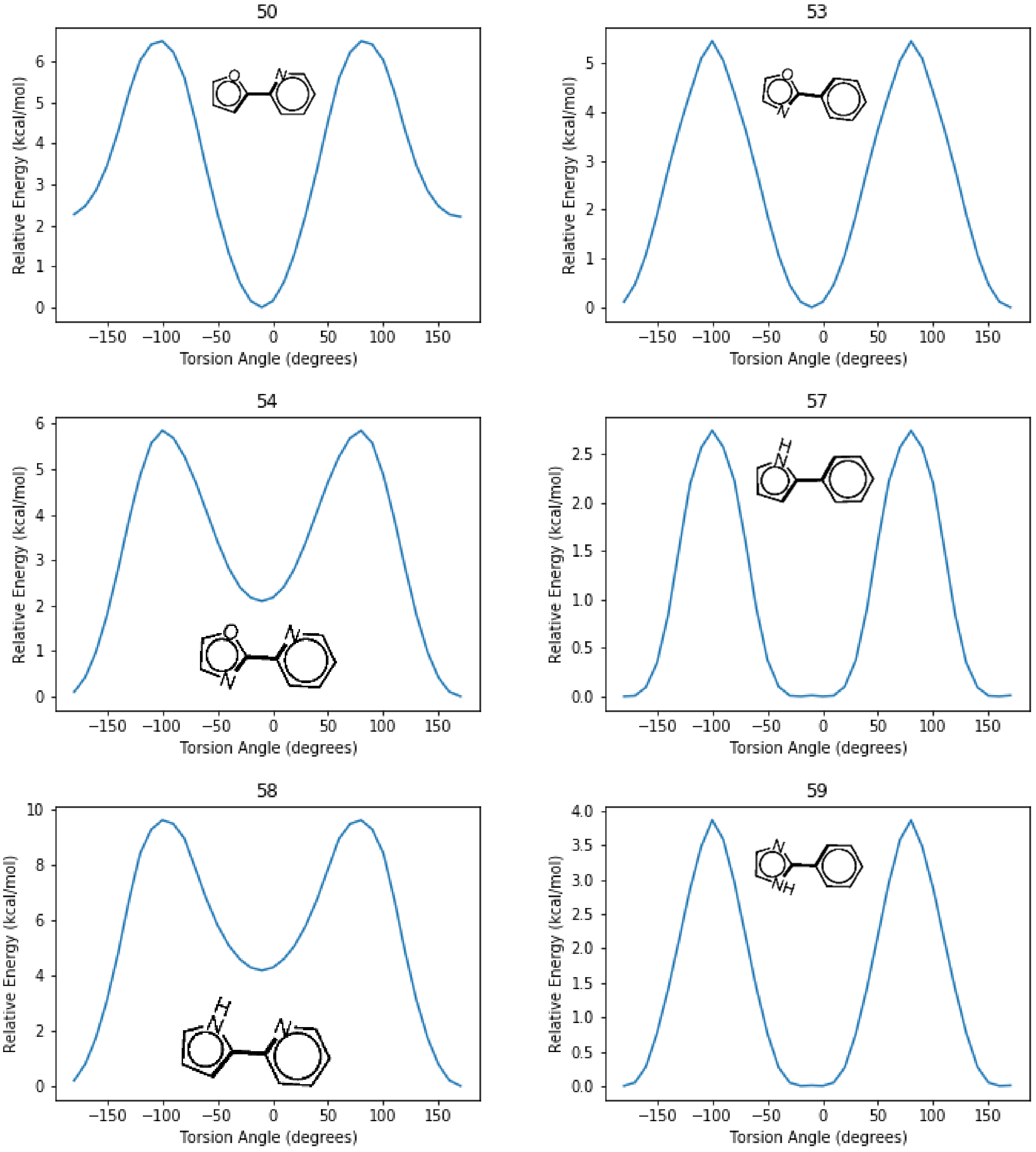
50
53
54
57
58
59
Relative Energy (kcal/mol)
Torsion Angle (degrees)

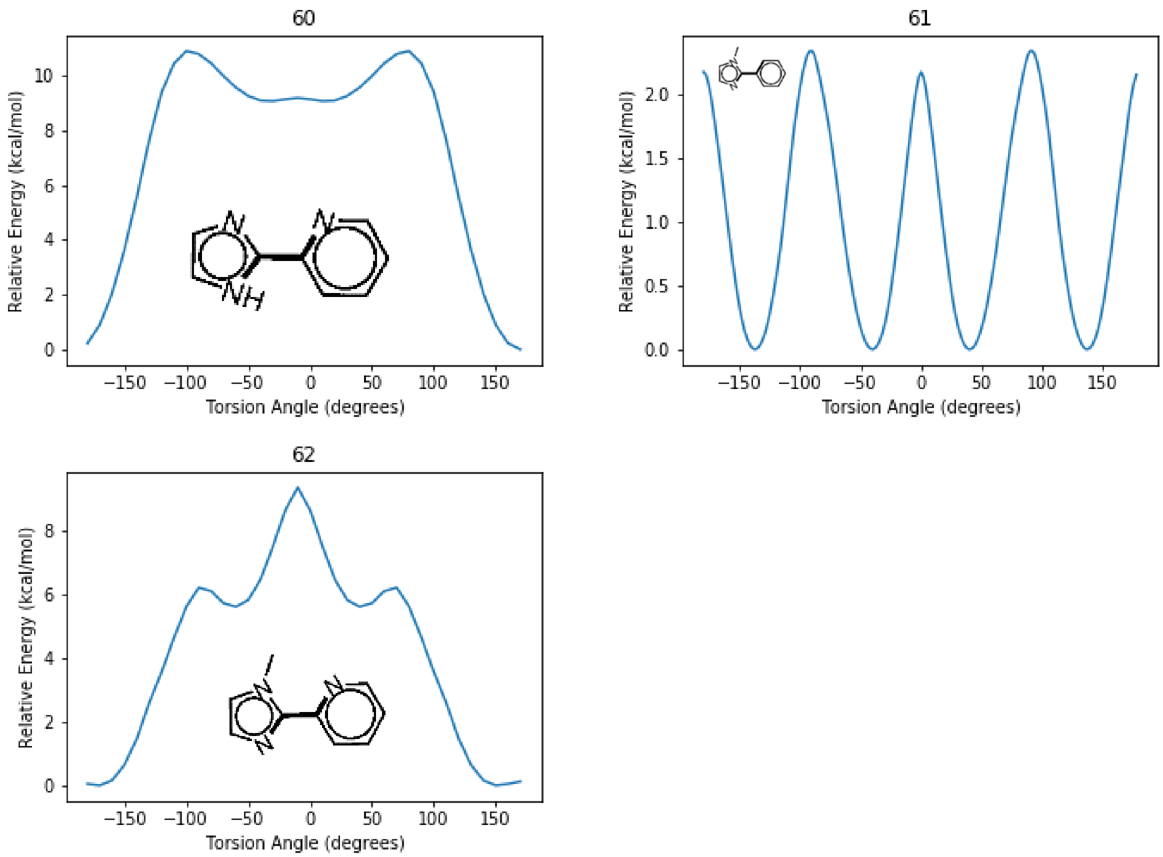


**Supplementary Figure 6:** Dihedrals scans with ANI-ccx used to compute the errors listed in Figure 4. The molecules are numbered according to reference 19, listing only atoms with C,H,N and O.

## Supplementary References:


(1) Liakos, D. G.; Sparta, M.; Kesharwani, M. K.; Martin, J. M. L.; Neese, F. Exploring the Accuracy Limits of Local Pair Natural Orbital Coupled-Cluster Theory. *J. Chem. Theory Comput.* **2015**, *11* (4), 1525–1539. https://doi.org/10.1021/ct501129s.

(2) Řezáč, J.; Riley, K. E.; Hobza, P. S66: A Well-Balanced Database of Benchmark Interaction Energies Relevant to Biomolecular Structures. *J. Chem. Theory Comput.* **2011**, *7* (8), 2427–2438. https://doi.org/10.1021/ct2002946.

(3) Friedrich, J.; Hänchen, J. Incremental CCSD(T)(F12*)|MP2: A Black Box Method to Obtain Highly Accurate Reaction Energies. *J. Chem. Theory Comput.* **2013**, *9* (12), 5381–5394. https://doi.org/10.1021/ct4008074.

(4) Liakos, D. G.; Neese, F. Improved Correlation Energy Extrapolation Schemes Based on Local Pair Natural Orbital Methods. *J. Phys. Chem. A* **2012**, *116* (19), 4801–4816. https://doi.org/10.1021/jp302096v.

(5) Hobza, P.; Sponer, J. Toward True DNA Base-Stacking Energies: MP2, CCSD(T), and Complete Basis Set Calculations. *J. Am. Chem. Soc.* **2002**, *124* (39), 11802–11808.

(6) Halkier, A.; Helgaker, T.; Jørgensen, P.; Klopper, W.; Olsen, J. Basis-Set Convergence of the Energy in Molecular Hartree–Fock Calculations. *Chem. Phys. Lett.* **1999**, *302* (X), 437–446. https://doi.org/10.1016/S0009-2614(99)00179-7.

(7) Helgaker, T.; Klopper, W.; Koch, H.; Noga, J. Basis-Set Convergence of Correlated Calculations on Water. *J. Chem. Phys.* **1997**, *106* (23), 9639–9646. https://doi.org/10.1063/1.473863.

(8) Neese, F.; Valeev, E. F. Revisiting the Atomic Natural Orbital Approach for Basis Sets: Robust Systematic Basis Sets for Explicitly Correlated and Conventional Correlated Ab Initio Methods? *J. Chem. Theory Comput.* **2011**, *7* (1), 33–43. https://doi.org/10.1021/ct100396y.

(9) Kesharwani, M. K.; Karton, A.; Sylvetsky, N.; Martin, J. M. L. The S66 Non-Covalent Interactions Benchmark Reconsidered Using Explicitly Correlated Methods Near the Basis Set Limit*. *Aust. J. Chem.* **2018**, 238–248. https://doi.org/10.1071/CH17588.

(10) Karton, A.; Daon, S.; Martin, J. M. L. W4-11: A High-Confidence Benchmark Dataset for Computational Thermochemistry Derived from First-Principles W4 Data. *Chem. Phys. Lett.* **2011**, *510* (4–6), 165–178. https://doi.org/10.1016/j.cplett.2011.05.007.

(11) Smith, J. S.; Isayev, O.; Roitberg, A. E. ANI-1: An Extensible Neural Network Potential with DFT Accuracy at Force Field Computational Cost. *Chem. Sci.* **2017**, *8* (4), 3192–3203. https://doi.org/10.1039/C6SC05720A.

(12) Smith, J. S.; Nebgen, B.; Lubbers, N.; Isayev, O.; Roitberg, A. E. Less Is More: Sampling Chemical Space with Active Learning. *J. Chem. Phys.* **2018**. https://doi.org/10.1063/1.5023802.

(13) Kingma, D.; Ba, J. Adam: A Method for Stochastic Optimization. *arXiv* **2014**, Preprint at https://arxiv.org/abs/1412.6980.

(14) Jupp, S.; Malone, J.; Bolleman, J.; Brandizi, M.; Davies, M.; Garcia, L.; Gaulton, A.; Gehant, S.; Laibe, C.; Redaschi, N.; et al. The EBI RDF Platform: Linked Open Data for the Life Sciences. *Bioinformatics* **2014**, *30* (9), 1338–1339. https://doi.org/10.1093/bioinformatics/btt765.

(15) Bento, A. P.; Gaulton, A.; Hersey, A.; Bellis, L. J.; Chambers, J.; Davies, M.; Krüger, F. A.; Light, Y.; Mak, L.; McGlinchey, S.; et al. The ChEMBL Bioactivity Database: An Update. *Nucleic Acids Res.* **2014**, *42* (D1), D1083–D1090. https://doi.org/10.1093/nar/gkt1031.

(16) Davies, M.; Nowotka, M.; Papadatos, G.; Atkinson, F.; van Westen, G.; Dedman, N.; Ochoa, R.; Overington, J. MyChEMBL: A Virtual Platform for Distributing Cheminformatics Tools and Open Data. *Challenges* **2014**, *5* (2), 334–337. https://doi.org/10.3390/challe5020334.

(17) Sellers, B. D.; James, N. C.; Gobbi, A. A Comparison of Quantum and Molecular Mechanical Methods to Estimate Strain Energy in Druglike Fragments. *J. Chem. Inf. Model.* **2017**, *57* (6), 1265–1275. https://doi.org/10.1021/acs.jcim.6b00614.

(18) Řezáč, J.; Riley, K. E.; Hobza, P. Extensions of the S66 Data Set: More Accurate Interaction Energies and Angular-Displaced Nonequilibrium Geometries. *J. Chem. Theory Comput.* **2011**, *7* (11), 3466–3470. https://doi.org/10.1021/ct200523a.

(19) Barron, J. T. Continuously Differentiable Exponential Linear Units. *arXiv* **2017**, Preprint at https://arxiv.org/abs/1704.07483.

(20) Peverati, R.; Zhao, Y.; Truhlar, D. G. Generalized Gradient Approximation That Recovers the Second-Order Density-Gradient Expansion with Optimized across-the-Board Performance. *J. Phys. Chem. Lett.* **2011**, *2* (16), 1991–1997. https://doi.org/10.1021/jz200616w.

(21) Luo, S.; Zhao, Y.; Truhlar, D. G. Validation of Electronic Structure Methods for Isomerization Reactions of Large Organic Molecules. *Phys. Chem. Chem. Phys.* **2011**, *13* (30), 13683. https://doi.org/10.1039/c1cp20834a.

(22) Falkowska, M.; Bowron, D. T.; Manyar, H. G.; Hardacre, C.; Youngs, T. G. A. Neutron Scattering of Aromatic and Aliphatic Liquids. *ChemPhysChem* **2016**, *17* (13), 2043–2055. https://doi.org/10.1002/cphc.201600149.
